# Application and Dosage

**Published:** 1914-03-21

**Authors:** 


### Application and Dosage.

Radium itself is used in two chief ways. It is spread out on a flat surface for application to superficial ulcers, inflammations, or growths; and it is enclosed in tubes for insertion into the various orifices of the body or into the actual substance of the tissues. Flat applicators are arranged so as to contain from two and a half to ten milligrams of radium per square centimetre of surface, varnished over to prevent any loss of the radium. Tubes are used as small as possible, and great stress is laid upon the necessity for packing them very tight. Radium emanation is also often used in tubes, compressed or condensed by liquid air, so as to get as much of it as possible, and thus the maximum of radio-activity concentrated into a small bulk, One of the great advantages of emanation is that, being a gas, it is always diffused absolutely uniformly throughout the containing vessel; and so the radiations from it are also given off uniformly in all directions. With solid radium salts, on the other hand, unless the packing is very tight and has been done with the greatest care so as to leave no empty space, the distribution of the radium may not be perfectly uniform, and, in consequence, one end of a tube may be emitting radiations of great strength while the other end emits almost none at all. In order to bring radiations to bear on parts of the body which are difficult of access—the larynx, for instance, or the tortuous recesses of a long sinus—many ingenious contrivances have been invented by radium workers. Every centre of radium therapy has its own particular methods of circumventing mechanical difficulties of this sort; and finality is, no doubt, very far from having been reached.

When the diseased part upon which radiations are to be directed is situated in a natural canal—the rectum, for instance, or the nose—it suffices to insert a tube of suitable shape and size containing such a quantity of the radio-active substance as is thought advisable, and to leave it for a certain length of time in the closest possible proximity to the diseased tissues. When the surface of the body is the site of the disorder, the application of a flat plate having a radium salt smeared uniformly over it is the usual method of radiating it. And this is also sometimes the plan adopted in treating diseases in internal organs, provided they are not too deeply situated in the interior. This method, however, has the drawback that the greater part of the radiation is wasted by being discharged into the outside air: only a minority of it can, even in the most favourable case, act upon the tissues. Accordingly, for many diseases in organs lying deep to the skin it is now the custom to implant tubes right into their midst; the tubes contain a radio-active substance, and by this device the whole of the radiation from them plays upon the diseased area. As a rule, this procedure requires the administration of an anæsthetic and a slight surgical operation. It must, of course, be carried out with the fullest and strictest antiseptic precautions by one accustomed to doing surgical operations.

Radium, and especially its emanation, are also administered by the mouth. Radium salts are believed to be irritant poisons if given in sufficient dosage, but are much too expensive to be wasted in this way. They are said to be excreted mainly in the fæces, and to a smaller degree by the kidneys. Radium emanation, however, is principally excreted through the lungs, as might be expected from its gaseous condition. This substance has been very largely tried by oral imbibition for various affections, with varying measures of success. Much of the emanation solution sold commercially contains only the most minute traces of radium emanation; and even *bona-fide* workers use strengths which differ enormously from one another. The solution must be swallowed fresh on account of the rapidity with which it decays. Another point which has to be borne in mind is the fact that this emanation when swallowed gives out $\alpha$-rays as well as $\beta$-rays, and that these may act upon the organs in which the emanation circulates. When external applications are used these $\alpha$-rays are stopped by the skin, and do not penetrate the deeper tissues. So that emanation solution may quite conceivably have totally different effects from those obtained by radium therapy applied from outside the body.

### Other Sources of Radio-Activity.

Before leaving the subject of the manner in which radium and its emanation are manipulated in order that they may cause radiations to impinge upon diseased parts, it must be added that there are other sources of radio-activity. Polonium, as will be gathered from what has already been said, is radio-active, and so is uranium. These are not much used therapeutically, but there is another radio-active substance which has been used a good deal, more especially in Germany: its name is mesothorium. Thorium itself is a metal which is used in certain arts, and is very slightly radio-active. One of its decomposition products is mesothorium, a substance much more radio-active than radium itself. Unfortunately this substance has never yet been obtained in anything approaching a state of purity. But when it is present in samples of radium the radio-activity of the mixture is much greater than that of radium itself, and rises steadily during the next two or three years to almost half as much again as its original radio-active power. Thenceforward the activity falls off, and in about ten or twenty years it reaches the original level. Finally, nothing but radium itself is left. From mesothorium 2 both $\beta$ and $\gamma$ rays are emitted, and some of the other thorium products give $\alpha$-rays also. Whether this substance, or some modification of it, will ever supplant radium is a matter which the future must decide.

### A Point for Hospital Committees.

The $x$-ray tube is, as has been stated, a source of radiations. At the present time there is no form of $x$-ray apparatus which can provide radiations of the exact character of those emitted from the radio-active substances. Some experts in radio-activity believe that it is only a matter of time before this will be remedied; and they look forward to a future when by mechanical means radiations will be made which will counterfeit in every respect those now provided by radium. Other experts do not hold this view, and believe that such a feat is impossible at present and for many years to come, if not, indeed, *in perpetuo*. This question is entirely distinct from that of the relative superiority of treatment by $x$-rays and of treatment by radium. The two questions have this much in common, that they both bear upon the financial aspect of the purchase of radium at its present inflated value. At present some of the highest authorities believe more in radium than in $x$-rays; others more in $x$-rays than in radium.

Among expert researchers in radium-therapy there are very wide divergences of view as to the best dosage of radium in the various diseased conditions for which it is used. At the very outset it is well to bear in mind that much of the radium now in use is more or less impure, and also that different salts of radium contain different percentages of the metal. But even after allowing for the discrepancies thus created, there are divergences of opinion so wide that they merely serve to show how incomplete our knowledge of radium-therapy is, and how useless are dogmatic assertions in the present stage of progress. An excellent illustration of this point is provided by the report of a meeting of the Berlin Medical Society early this year. Speaker A preferred to use 50 to 100 milligrams, and denounced 300 mg. doses as dangerous. B eulogised 150 mg. as a commencement, and said that smaller doses may actually make the patient worse instead of better. C took exactly the opposite view, favouring very small doses and deprecating large ones. D was enthusiastic on the benefits of 500 mg., and thought his results were much better than those of his more timorous brethren. E was sceptical about the whole matter, and inclined to regard radium as a useless method of treatment! Now all these speakers were men of high reputations. When experts thus agree to differ—and views as contradictory prevail in England too—the uselessness of definite rules as to the dosage of radium is self-evident. Hospital committees will do well to leave their radium experts a free hand for the present.

